# An Evaluation of MAPIA in Michigan as an Ante-Mortem Supplemental Test for Use in Suspect Tuberculosis Cattle

**DOI:** 10.1155/2012/674368

**Published:** 2012-04-10

**Authors:** Scott D. Fitzgerald, Heather A. Grodi, John B. Kaneene

**Affiliations:** ^1^Department of Pathobiology and Diagnostic Investigation, College of Veterinary Medicine and the Diagnostic Center for Population and Animal Health, Michigan State University, Lansing, MI 48910, USA; ^2^Center for Comparative Epidemiology, College of Veterinary Medicine, Michigan State University, East Lansing, MI 48824, USA

## Abstract

The objective of this study was to make use of bovine tuberculosis suspect cattle from the state of Michigan to validate a multiantigen print immunoassay for use on sera to serve as an improved supplementary ante-mortem test to increase specificity of current tuberculosis testing methods. Over a 27-month period, 234 sera were collected and tested by MAPIA method, which was evaluated using four different interpretation criteria. These results were subsequently compared to final mycobacterial culture and PCR results obtained by the National Veterinary Services Laboratories, Ames, IA, which served as the true indicator of the cattle's tuberculosis infection status. This study indicates that an interpretation criterion which includes 3 or more positive reactions to the 11 different mycobacteria antigens utilized provided both an acceptable sensitivity (69.39%) and a high specificity (90.27%). This MAPIA technique shows potential for eventual application as a supplementary ante-mortem tuberculosis serologic test following one of the various current or soon-to-be-approved whole herd screening assays as part of a tuberculosis eradication program.

## 1. Introduction

Since the state of Minnesota has been reclassified as bovine tuberculosis accredited free in October, 2011, there are few USA states remaining which have endemic bovine tuberculosis in either its domestic cattle or a free-ranging wildlife reservoir host [1]. Presently, only Michigan and California are not classified as state-wide bovine tuberculosis accredited-free states. Therefore, these states are the logical locations in which to conduct trials on new or alternative bovine tuberculosis assays.

 The ongoing decline in bovine tuberculosis in the USA, coupled with the ongoing economic recession, has led the USDA to reevaluate its current approaches for bovine tuberculosis surveillance and eradication [2]. The USDA will now be increasing the options for managing tuberculosis-infected herds, and developing alternative control strategies other than whole-herd depopulation. As part of this process, the USDA is also accelerating development of new diagnostic tests for ante-mortem cattle testing. The rapid test or lateral flow assay is one of those new diagnostic tests for use on bovine serum which is currently in final stages of validation prior to USDA licensure and market introduction [3]. One of the needs for this new assay is development of a supplemental assay to be used as a followup on cattle which are considered as suspects or reactors to the initial rapid test, much as the comparative cervical test was used for decades as a supplemental assay on cattle which reacted to the caudal tail fold test.

 Over the last three years, our laboratory has been standardizing, validating, and applying the multiantigen print immunoassay (MAPIA) which was initially developed by Lyashchenko and others [4]. This ante-mortem serum-based Western blot assay utilizes several of the specific antigens which will be included in the rapid test, therefore making it a logical choice as the supplemental assay. All current approved ante-mortem cattle tuberculosis tests (caudal fold test, comparative cervical test, gamma-interferon assay) are all based on testing the cellular immune response. The MAPIA is a serologic assay which tests the humoral immune response [5]. Currently, Michigan is still in the process of dealing with bovine tuberculosis endemic in its wild white-tailed deer, which annually spills over into multiple domestic cattle herds each year, and so Michigan is a natural location in which to investigate the specific capabilities of this assay under field conditions, utilizing sera from both suspect and exposed cattle. Furthermore, while current specificity of the approved caudal fold test and comparative cervical assays have always been reported to be in the high 90 percentiles, we found our diagnostic laboratory was processing 10, 20, even 30 or more indemnified suspect cattle to obtain a single positively infected individual [5]. Our goal in this study was to try to develop and evaluate a supplemental ante-mortem assay which could significantly reduce the false positives (i.e., increase specificity) that current screening methods were producing, while still maintaining a high enough sensitivity to move the detection and eradication process for bovine tuberculosis forward.

## 2. Materials and Methods

### 2.1. Cattle

Sera were collected from live cattle submitted to the Diagnostic Center for Population and Animal Health, Michigan State University, by the Michigan Department of Agriculture for tuberculosis testing as part of their ongoing tuberculosis eradication program. Using the USDA Uniform Methods and Rules, these cattle were classified as caudal fold test suspects or reactors (CFT Suspects), comparative cervical test suspects (CCT Suspects), gamma-interferon assay suspects (IFN-*γ* Suspects), gamma interferon assay failed (IFN-*γ* Failed), reactor cattle not otherwise specified to the initial assay utilized (Reactor NS), traceback cattle originating from a tuberculosis positive herd (Traceback), or cattle exposed to a another known positive animal (Exposed) [6]. Cattle have generally been field testing using tuberculin between 3 and 6 weeks prior to their submission to the Diagnostic Center, although some cattle my take as long as 2 to 4 months after field testing before being submitted. According to current methods, neither traceback nor exposed cattle are required to undergo any ante-mortem tuberculosis testing prior to removal and necropsy/slaughter. Following blood collection, cattle were humanely euthanized and underwent complete necropsy including collection of all major lymph nodes from their head, thorax, and abdomen for routine histopathology, acid-fast staining, and mycobacterial culture, and PCR at the National Veterinary Services Laboratories, Ames, IA [7]. The culture and PCR results provided by NVSL served as the definitive gold standard as to whether cattle were infected with *Mycobacterium bovis* or not. These cattle were all sampled between July 1, 2009, and September 30, 2011. Exceptions to this included two cattle previously sampled on April 30, 2001, which were in an advanced state of tuberculosis and served as known positives for initial assay standardization; 20 known positive sera from other states (Nebraska, Texas, South Dakota, Colorado, and Indiana) purchased from the USDA Tuberculosis Serum Bank to increase the number of positive samples in the study; and 11 beef cattle with gross lesions from Michigan which went directly to slaughter but for which the sera were collected and the same lymph node histopathology, culture, and PCR testing were performed at NVSL.

### 2.2. MAPIA

The MAPIA assay was modified slightly from the technique previously described [4, 8]. Briefly, sera were stored frozen at −20°C, thawed, and diluted 1 : 20. Eleven antigens were diluted to 50 *μ*g/mL in PBS, then applied by a semiautomated airbrush-printing device (Linomat 5, Camag Scientific Inc., Wilmington, NC, USA) onto nitrocellulose membranes Protran Nitrocellulose Membrane (Whatman, Dassel, Germany) in eleven 12 cm long parallel strips. A blue stain (Coomassie Blue R350, GE Healthcare, Piscataway, NJ, USA) was also applied to the membranes to serve as an indicator of the correct up-side of the membrane, to indicate the bottom end of the membrane, and to serve as a standard reference on each membrane with which to compare the strength of the antigen-antibody reactions. After drying, the membranes were cut perpendicular to the antigen strips at approximately 4 mm widths, creating test strips with 11 different antigen lanes plus the Coomassie Blue band. Strips were blocked for 1 hr with 1% skim milk in phosphate buffered saline (PBS) with 0.05% Tween (Kirkegaard and Perry Laboratories Inc., Gaithersburg, MD, USA), incubated with each serum sample diluted 1 : 20 in PBS (Kirkegaard and Perry Laboratories) for 2 hrs, washed three times with PBS, reacted with Protein G (Sigma-Aldrich, St. Louis, MO, USA) for 1 hr, washed 3 times, and finally reacted with 3,3′,5,5′-tetramethylbenzidine peroxidase (TMB 1-Component Membrane Peroxidase Substrate, Kirkegaard and Perry Laboratories) for 5 min. Strips were rinsed in cold water 3 times to stop the reaction and then air-dried overnight before being read for results. Strip results were read by unaided eye as either negative, weak positive if the line of reactivity was thin and less intense than the control band of Coomassie Blue stain, or strong positive if the line of reactivity was as thick and of similar intensity as the Coomassie Blue stain band.

### 2.3. Antigens

Antigens selected for use had been previously reported as regularly occurring in *Mycobacterium bovis* or *M. tuberculosis*, or as inducing significant antibody responses in cattle with bovine tuberculosis infections [9]. These recombinant antigens included ESAT-6 [10, 11] (Statens Serum Institut, Copenhagen, Denmark); ESAT-6/CFP10 fusion protein [12] and MPB83 [13, 14] (provided by a collaborator at National Animal Disease Center, Ames, IA); Acr1 [15], 38 kDa [16], 45 kDa [17], Ag85B [18], GroES [19] (all from TB Vaccine Testing and Research Materials Contract, Colorado State University, Fort Collins, CO, USA), and MPB59, MPB64, and MPB70 [20] (provided by a collaborator at Agri-Food and Biosciences Institute, Belfast, Northern Ireland).

### 2.4. Statistical Analysis

 MAPIA results were interpreted using the NVSL mycobacterial culture and PCR results as the true tuberculosis status of the tested cattle. Four different criteria for assay interpretation were developed as follows. Criterion one is a positive reaction to any single antigen; criterion two is positive reactions to any two antigens; criterion three is positive reactions to any 3 or more antigens; and criterion four is a strong positive reaction to any single antigen. Each of these was then calculated for sensitivity, specificity, and positive or negative predictive values. In addition, each individual antigen was evaluated for sensitivity, specificity, positive and negative predictive values.

## 3. Results

Of the 234 cattle tested for this study, 49 were true positive cattle based on mycobacterial positive cultures and PCR positive results for *M. bovis* at the NVSL, Ames, IA. The authors acknowledge that some positive cattle in very early stages of infection may not have exhibited gross or histologic lesions of tuberculosis and may not have been detected by current culture methods; however, for the purposes of this study true positive cattle must have positive mycobacterial culture or PCR results. The remaining 185 cattle were true negatives based on negative PCR and culture results.

Four criteria were used to evaluate the MAPIA results as previously described in the methods. Of these, criteria 1, 2, and 3 all provided good sensitivities compared to the true positive status of the individual cattle tested (see Table 1). But criterion 3 provided by far the best specificity of 90.27%, which was significantly better than either criterion 1 or 2. In addition, using positive predictive value, criterion 3 was the best criteria correctly identifying nearly two-thirds of all true positive animals as positive, while both criteria 1 and 2 had significantly lower positive predictive values. Therefore, for our current situation in Michigan, criterion 3 proved to be the best method for using the MAPIA assay.

Table 2 illustrates how criterion 3 correlates with each of the antemortem categories of cattle (CFT suspects, CTT suspects, IFN-*γ* suspect, etc.). This criterion correlates best with CFT suspect/reactors and IFN-*γ* suspects, while it does not correlate as well with CCT suspects. There were no true positive cattle in the traceback and exposed cattle, so the method cannot be meaningfully evaluated for its performance in these categories. Nor were cattle in this study included in Table 2 if their initial method of field testing (CFT, CCT, IFN-*γ*) were not known.

Table 3 compares the sensitivity, specificity, and positive and negative predictive values for each of the 11 specific antigens used in our MAPIA assay. While ESAT-6 and the ESTA-6/CFP10 fusion protein had the highest sensitivities (83.67% and 87.76% resp.), these two antigens also demonstrated the lowest specificities (47.57% and 70.27% resp.). The other 9 remaining antigens tested exhibited significantly lower sensitivities, but also uniformly higher specificities. 

## 4. Discussion

MAPIA assay in our laboratory, compared to known positive and negative bovine tuberculosis infected cattle primarily from the state of Michigan, offers promise as a supplemental test. The MAPIA assay requires some specialized equipment, some moderately expensive reagents, and approximately 4.5 hours of time to run. The time, equipment, cost all make this assay less than optimal for whole herd screening. But as a follow-up or supplemental test, especially for the CFT, this assay when interpreted using criterion 3 offers high sensitivity (84.62%) and high specificity (92.31%). This in turn can lead to large monetary savings to state and federal agencies by significantly reducing the total number of cattle indemnified, transported to necropsy facilities, and undergoing extensive post-mortem testing.

 For example on the cost savings, and using the 234 cattle included in this study as an example, if we had run the MAPIA before sacrificing these cattle, only 78 would have been considered suspects and sent to necropsy using criterion 3. The MAPIA costs between $100 and $150 to run including reagents, technician time, and so forth. Multiplying 234 cattle sera by $150 results in an additional cost of doing this supplemental test of $35,100. Now we calculate the cost of sacrificing those other 156 cattle which the MAPIA would have classified as not suspects. Maximum indemnity in Michigan is currently $3500 per cow; we will take $1750 as an estimate at the average indemnity cost of a cow. Add in the state of Michigan paying for a 4-hour hauling charge from the endemic tuberculosis area to the laboratory, estimated at $150 per cow, the Diagnostic Laboratory charge of $250 per cow for full tuberculosis surveillance workup, and the additional charges incurred by the USDA for mycobacterial culture, PCR, and histopathology on the harvested lymph nodes estimated at $450 per cow. This totals to an average cost of $2,600 per cow. Multiplying 156 cattle by the average cost of $2,600 we get an additional cost of $405,600. The extra cost of $35,100 for the additional MAPIA testing, is more than offset by the additional cattle costs of $405,600, resulting in a net savings of $370,500. This is not a perfect result as 12 true positive cattle would have remained on their farms since MAPIA criterion 3 did not call them positive. However, this assay can be interpreted by various criteria. Criterion 1 would have only missed one positive cattle, and criterion 2 would have only missed three positive cattle; but both criteria would have required the sacrifice of additional cattle, resulting in lower total monetary savings. Remember that each state could select the MAPIA interpretation which makes the most sense for their situation. In Michigan, you might reasonably select criterion 1 to miss the lowest number of positive cattle since tuberculosis is present in both the cattle and wildlife populations. Most of the US states are free of tuberculosis and could select a more specific interpretation criterion by which to use the MAPIA. The interpretation could also be adjusted depending on the area the cattle were in (accredited free, modified accredited free, etc.), or if the farm was known to have other currently infected animals or if the farm had previously contained infected animals then more sensitive criterion could be used.

 Several researchers have indicated that ESAT-6 or ESAT-6/CFP10 are among the best antigens to be used for ante-mortem serologic testing for tuberculosis [9–12, 21]. Our data indicates that these two antigens do detect the highest percentages of true positive tuberculous cattle and result in the highest sensitivity. However, our results also show relatively low specificity for both antigens due to many false positives. These antigens may not be as specific for *Mycobacterium bovis* as previously believed. Alternatively, they are secreted so early in the immune response that they may increase rapidly following the intradermal injection of tuberculin utilized in the CFT or CCT tests, therefore resulting in false positives. One recent study actually documented a significant boost in BCG-vaccinated cattle following the use of the intradermal tuberculin test, resulting in increased immunoglobulin levels of eight different mycobacterial antigens when measured by the MAPIA [9]. Whatever the reason, all 9 of the other antigens utilized in this study had significantly higher specificities (ranging from 88.65% up to 100%), but also significantly lower sensitivities (ranging from 2.04% to 48.98%). It is interesting to note of the 9 antigens evaluated other than ESAT-6 and ESTA-6/CFP10, the two antigens showing the highest sensitivity were 45 kDA and MPB70 (both had sensitivities of 48.98%). MPB70 has been previously shown to have high sensitivity in detecting tuberculous cattle in surveys conducted in a number of countries [21]. Therefore, by using a criterion for MAPIA interpretation which combines multiple antigen reactions, one gains increased specificity while maintaining high overall sensitivity.

 Looking for stronger positive reactions as in criterion 4 in the MAPIA did not result in increased sensitivity, but actually decreased sensitivity (59.18%) to the lowest of all four criteria used. This may reflect the less important role of antibody response in tuberculosis infection compared to cell-mediated immunity; therefore, infected cattle may not necessarily develop the highest antibody response against mycobacterial antigens. For whatever reason, strength of antibody-antigen reaction was not highly correlated with true tuberculosis status. For this reason, the authors chose to simplify the reading and reporting of the assay to three simple responses, negative, weak, or strong and not to include quantitative optical density measurements for each antigen-antibody reaction within the MAPIA assay as in some previous studies [4].

 One interesting side note is how this MAPIA assay was performed in cattle which were negative for *M. bovis*, but from which environmental mycobacteria outside the *M. tuberculosis*-group were isolated. Only four cattle out of 234 were culture positive for either *M. avium* (2 isolates) or non-*M. tuberculosis* group (2 isolates), not otherwise specified. So the numbers are too low to make any generalizations about how the MAPIA assay performs. However, these 4 cattle were uniformly interpreted negative on MAPIA testing by criteria 2, 3, and 4; while 2 of 4 cattle (both non-*M. tuberculosis* group individuals) were interpreted positive by criterion 1. This reinforces the value to utilizing an interpretation criterion that includes more than one positive response as a method to increase the test specificity.

 Ideally, our evaluation of MAPIA will continue, with testing of additional known positive and negative cattle. In addition, if this assay is to someday be approved by USDA as a supplemental test, we would ideally like to run all our banked sera samples by the rapid test—lateral flow assay. That assay—as previously stated—is in the approval process for validation and licensure for use in the US [3]. Since that assay utilizes several of the same antigens as the MAPIA, and is an ante-mortem serologic assay, it would be important to compare these two assays performance on the same set of sera. However, since the rapid test is not yet approved, and therefore not commercially available, we have been unable to complete this important validation step to date.

The MAPIA assay requires some specialized equipment, some moderately expensive reagents, and approximately 4.5 hours of time to run. However, with the proper application of methods, selection of test antigens, and correct interpretation criteria, it shows remarkable promise for use as limited supplemental test following initial ante-mortem field screening tests. While the MAPIA is currently too expensive to use as a primary screening test for tuberculosis in cattle, its use to either increase sensitivity or specificity depending on the specific needs of the state, area or farm, when used as a supplemental test, could prove valuable as an epidemiologic tool, and potentially result in significant cost savings for the national tuberculosis eradication program.

## Figures and Tables

**Table 1:** Sensitivity, specificity, positive predictive value, and negative predictive value of the MAPIA test, using four different interpretation criteria.

MAPIA test criteria*	Results	True status		Test performance		Predictive value	
		Positive (*n* = 49)	Negative (*n* = 185)	Sensitivity	Specificity	Positive	Negative
(1) Any positives	Positive	48	120	97.96	35.14	28.57	98.48
	Negative	1	65				
(2) Two positives	Positive	46	52	93.88	71.89	46.94	97.79
	Negative	3	133				
(3) Three positives	Positive	34	18	69.39	90.27	65.38	91.76
	Negative	15	167				
(4) Any strong positive	Positive	29	22	59.18	88.11	56.86	89.07
	Negative	20	163				

*Test criteria.

(1) Any positives: weak or strong positive reaction to at least one antigen

(2) Two positives: weak or strong positive reaction to at least two antigens

(3) Three or more positives: weak or strong positive reaction to at least three antigens

(4) Any strong positive: strong positive reaction to at least one antigen.

**Table 2:** Sensitivity, specificity, positive predictive value, and negative predictive value of a weak or strong positive reaction to at least three antigens (criterion 3) in the MAPIA test, by different antecedent tests.

MAPIA test criteria*	Results	True status		Test performance		Predictive value	
		Positive (*n* = 49)	Negative (*n* = 185)	Sensitivity	Specificity	Positive	Negative
CFT suspect/reactor	Positive	11	2	84.62	92.31	84.62	92.31
	Negative	2	24				
CCT suspect	Positive	1	2	50.0	90.91	33.33	95.24
	Negative	1	20				
IFN-*γ* suspect	Positive	12	10	70.59	81.82	54.54	90.0
	Negative	5	45				
Traceback	Positive	0	4	—	80.0	—	100.0
	Negative	0	16				
Exposed	Positive	0	0	—	100.0	—	100.0
	Negative	0	58				

**Table 3:** Sensitivity, specificity, positive predictive value, and negative predictive value of weak or strong positive reactions to individual antigens used in MAPIA.

Antigen	Results	True status		Test performance		Predictive value	
		Positive (*n* = 49)	Negative (*n* = 185)	Sensitivity	Specificity	Positive	Negative
ESAT-6	Positive	41	97	83.67	47.57	29.71	91.67
	Negative	8	88				
ESAT-6/CFP10	Positive	43	55	87.76	70.27	43.88	95.59
	Negative	6	130				
Acr1	Positive	13	9	26.53	95.14	59.09	83.02
	Negative	36	176				
38 kDa	Positive	2	1	4.08	99.46	66.67	79.65
	Negative	47	184				
45 kDa	Positive	24	21	48.98	88.65	53.33	86.77
	Negative	25	164				
Ag85B	Positive	5	3	10.20	98.38	62.50	80.53
	Negative	44	182				
GroES	Positive	14	8	28.57	95.68	63.64	83.49
	Negative	35	177				
MPB83	Positive	3	0	6.12	100.0	100.0	80.09
	Negative	46	185				
MPB59	Positive	1	0	2.04	100.0	100.0	79.40
	Negative	48	185				
MPB64	Positive	2	0	4.08	100.0	100.0	79.74
	Negative	47	185				
MPB70	Positive	24	3	48.98	98.38	88.89	87.92
	Negative	25	182				

## References

[1] Hahn B USDA approves Minnesota TB free status. http://www.bah.state.mn.us/tb/releases/tbnr2011-10-05.html.

[2] A new approach for managing bovine tuberculosis: veterinary services’ proposed action plan. http://www.aphis.usda.gov/animal_health/animal_disease/tuberculosis/downloads/tb_concept_paper.pdf.

[4] Lyashchenko KP, Singh M, Colangeli R, Gennaro ML (2000). A multi-antigen print immunoassay for the development of serological diagnosis of infectious diseases. *Journal of Immunological Methods*.

[5] de la Rua-Domenech R, Goodchild AT, Vordermeier HM, Hewinson RG, Christiansen KH, Clifton-Hadley RS (2006). Ante mortem diagnosis of tuberculosis in cattle: a review of the tuberculin tests, *γ*-interferon assay and other ancillary diagnostic techniques. *Research in Veterinary Science*.

[6] Bovine tuberculosis eradication: uniform methods and rules, effective January, 2005.

[7] Fitzgerald SD, Bolin SR, Boland KG (2011). Overt *Mycobacterium avium* subsp. *paratuberculosis* infection an infrequent occurrence in archived tissue from false TB reactor cattle in Michigan, USA. *Veterinary Medicine International*.

[8] O’Brien DJ, Schmitt SM, Lyashchenko KP (2009). Evaluation of blood assays for detection of *Mycobacterium bovis* in white-tailed deer (*Odocoileus virginianus*) in Michigan. *Journal of Wildlife Diseases*.

[9] Lyashchenko K, Whelan AO, Greenwald R (2004). Association of tuberculin-boosted antibody responses with pathology and cell-mediated immunity in cattle vaccinated with *Mycobacterium bovis* BCG and infected with *M. bovis*. *Infection and Immunity*.

[10] Pollock JM, Andersen P (1997). Predominant recognition of the ESAT-6 protein in the first phase of infection with *Mycobacterium bovis* in cattle. *Infection and Immunity*.

[11] Pollock JM, Andersen P (1997). The potential of the ESAT-6 antigen secreted by virulent mycobacteria for specific diagnosis of tuberculosis. *Journal of Infectious Diseases*.

[12] Van Pinxteren LAH, Ravn P, Agger EM, Pollock J, Andersen P (2000). Diagnosis of tuberculosis based on the two specific antigens ESAT-6 and CFP10. *Clinical and Diagnostic Laboratory Immunology*.

[13] Mcnair J, Corbett DM, Girvin RM, Mackie DP, Pollock JM (2001). Characterization of the early antibody response in bovine tuberculosis: MPB83 is an early target with diagnostic potential. *Scandinavian Journal of Immunology*.

[14] Wiker HG, Lyashchenko KP, Aksoy AM (1998). Immunochemical characterization of the MPB70/80 and MPB83 proteins of *Mycobacterium bovis*. *Infection and Immunity*.

[15] Kennaway CK, Benesch JLP, Gohlke U (2005). Dodecameric structure of the small heat shock protein Acr1 from *Mycobacterium tuberculosis*. *Journal of Biological Chemistry*.

[16] Pollock JM, Douglas AJ, Mackie DP, Neill SD (1995). Peptide mapping of bovine T-cell epitopes for the 38 kDa tuberculosis antigen. *Scandinavian Journal of Immunology*.

[17] Romain F, Laqueyrerie A, Militzer P (1993). Identification of a *Mycobacterium bovis* BCG 45/47-kilodalton antigen complex, an immunodominant target for antibody response after immunization with living bacteria. *Infection and Immunity*.

[18] Oner F, Tian XX, Son K, Klegerman ME, Groves MJ (1994). Characterization of the antigen 85 complex fibronectin-binding proteins derived from aged culture filtrates of *Mycobacterium bovis* BCG, Tice substrain. *Microbios*.

[19] Cobb AJ, Frothingham R (1999). The GroES antigens of *Mycobacterium avium* and *Mycobacterium paratuberculosis*. *Veterinary Microbiology*.

[20] Fifis T, Costopoulos C, Radford AJ, Bacic A, Wood PR (1991). Purification and characterization of major antigens from a *Mycobacterium bovis* culture filtrate. *Infection and Immunity*.

[21] Aagaard C, Govaerts M, Meikle V (2006). Optimizing antigen cocktails for detection of *Mycobacterium bovis* in herds with different prevalences of bovine tuberculosis: ESAT6-CFP10 mixture shows optimal sensitivity and specificity. *Journal of Clinical Microbiology*.

